# Evaluation of ^18^F-fluorothymidine positron emission tomography ([^18^F]FLT-PET/CT) methodology in assessing early response to chemotherapy in patients with gastro-oesophageal cancer

**DOI:** 10.1186/s13550-016-0234-3

**Published:** 2016-11-16

**Authors:** R. Sharma, P. Mapelli, G. B. Hanna, R. Goldin, D. Power, A. Al-Nahhas, S. Merchant, R. Ramaswami, A. Challapalli, T. Barwick, E. O. Aboagye

**Affiliations:** 1 Department of Surgery and Cancer, Imperial College London, London, UK; 2 Department of Gastro-Oesophageal Surgery, Imperial College Healthcare NHS Trust, London, UK; 3 Department of Pathology, Imperial College Healthcare NHS Trust, London, UK; 4 Department of Oncology, Imperial College Healthcare NHS Trust, London, UK; 5 Department of Radiology/Nuclear Medicine, Imperial College Healthcare NHS Trust, London, UK; 6 Medical Oncology and Clinical Pharmacology, Imperial College London, Hammersmith Campus, Du Cane Road, W12 0HS London, UK

**Keywords:** Gastro-oesophageal cancer, [18 F]FLT-PET, Chemotherapy, Early response, RECIST 1.1

## Abstract

**Background:**

3’-Deoxy-3’-[^18^F]fluorothymidine ([^18^F]FLT) PET has limited utility in abdominal imaging due to high physiological hepatic uptake of a tracer. We evaluated [^18^F]FLT-PET/CT combined with a temporal-intensity information-based voxel-clustering approach termed kinetic spatial filtering (KSF) to improve tumour visualisation in patients with locally advanced and metastatic gastro-oesophageal cancer and as a marker of early response to chemotherapy.

Dynamic [^18^F]FLT-PET/CT data were collected before and 3 weeks post first cycle of chemotherapy. Changes in tumour [^18^F]FLT-PET/CT variables were determined. Response was determined on contrast-enhanced CT after three cycles of therapy using RECIST 1.1.

**Results:**

Ten patients were included. Following application of the KSF, visual distinction of all oesophageal and/or gastric tumours was observed in [^18^F]FLT-PET images. Among the nine patients available for response evaluation (RECIST 1.1), three patients had responded (partial response) and six patients were non-responders (stable disease). There was a significant association between Ki-67 and all baseline [^18^F]FLT-PET parameters. Area under the curve (AUC) from 0 to 1 min was associated with treatment response.

**Conclusions:**

The results of this study indicate that application of the KSF allowed accurate visualisation of both primary and metastatic lesions following imaging with the proliferation marker, [^18^F]FLT-PET/CT. However, [^18^F]FLT-PET uptake parameters did not correlate with response. Instead, we observe significant changes in tracer delivery following chemotherapy suggesting that further [^18^F]FLT-PET/CT studies in this tumour type should be undertaken with caution.

## Background

Chemotherapy is the mainstay of therapy for patients both with locally advanced and metastatic gastro-oesophageal cancers (GOCs) [1, 2]. In patients with locally advanced disease, the aim of neoadjuvant chemotherapy is to downstage the tumour to enable complete surgical resection, whilst in the metastatic setting, the overall goal is palliation. In both clinical scenarios, accurate and sensitive evaluation of tumour response is critical as combination chemotherapy is not without significant side-effects. Therefore, there is a need for early assessment of tumour response to minimise patient exposure to potentially toxic treatment regimens, especially in those patients unlikely to benefit.

The MUNICON I trial conducted in patients with tumours of the oesophagogastric junction illustrated that early metabolic response assessment with [^18^F]fluorodeoxyglucose ([^18^F]FDG-PET) guides therapy allocation following 2 weeks of therapy [3]. However, [^18^F]FDG-PET may not clearly distinguish complete response and residual disease or between residual disease and post-treatment inflammation. Moreover, [^18^F]FDG-PET has variable sensitivity in assessing gastric cancer [4, 5]. There is a need therefore to develop more specific tracers for both predicting and monitoring efficacy of chemotherapy.

3’-Deoxy-3’-[^18^F]fluorothymidine ([^18^F]FLT) is a surrogate marker of proliferation, with uptake reflecting the activity of thymidine kinase (TK-1), the key enzyme in the salvage pathway for thymidine monophosphate production; its expression correlating with the *S* phase of the cell cycle [6]. Although it has been reported that [^18^F]FLT-PET has higher sensitivity than [^18^F]FDG in detection of gastric cancer, in general, the level of uptake in tumours is lower with [^18^F]FLT than [^18^F]FDG-PET [7, 8]. The main limitation of [^18^F]FLT in imaging upper abdominal tumours stems from high background activity in the liver due to glucuronidation of [^18^F]FLT by normal hepatocytes, making visualisation of gastric and gastro-oesophageal junction tumours challenging [9, 10]. However, as [^18^F]FLT is a surrogate marker of DNA synthesis, it is more specific for malignancy and less susceptible to inflammatory changes compared with [^18^F]FDG. Therefore, it may be a better imaging biomarker for both prognostification and response evaluation in GOCs.

Recently, we devised a new temporal-intensity information-based voxel-clustering approach—kinetic spatial filter (KSF)—for removing normal, physiological [18 F]FLT uptake by the liver and to enable visualisation of specific uptake (i.e., uptake due to phosphorylation) in liver metastases [11]. Briefly, the KSF compares, on a voxel by voxel basis, the time activity curves (TACs) of the image with the TAC of predefined tissue classes. We therefore conducted this pilot, exploratory study to assess whether [^18^F]FLT-PET/CT in combination with the KSF could be used to improve visualisation and permit early prediction of clinical response following one cycle of chemotherapy in patients with locally advanced or metastatic GOC.

## Methods

### Patients

Ten patients with a histological diagnosis of locally advanced or metastatic adenocarcinoma of stomach or lower third of the oesophagus were recruited. Inclusion criteria were: patients suitable for chemotherapy, ECOG performance status ≤2 and at least one (primary or metastatic) lesion ≥20 mm outside the liver, as assessed by contrast CT chest and abdomen. All patients included in the study had blood counts and liver and renal functions acceptable for chemotherapy. The study was approved by the local ethics committee. All patients gave fully informed consent according to the Declaration of Helsinki guidelines. The administration of radioactivity for the PET scans was approved by the Administration of Radioactive Substances Advisory Committee, UK.

### Imaging protocol

[^18^F]FLT was manufactured according to standard protocols. All patients were scanned on a Siemens Biograph 64-slice PET/CT scanner. Baseline [^18^F]FLT-PET/CT was performed within a week prior to start of chemotherapy. Post-treatment PET/CT was performed within 3 weeks after the start of the first cycle of chemotherapy. In all cases, the primary tumour, regional lymph nodes and liver were imaged in a single thoracic/abdominal bed position. Patient positioning was followed by a CT scan (300 mA, 120 kVp, 1.35 pitch, 0.8 s/rotation) for both attenuation correction and co-registration with PET images, to allow good anatomical visualisation and localisation of [^18^F]FLT activity. [^18^F]FLT, mean (±SD) 208.2 ± 10.4 MBq, was injected as a bolus intravenously, and a dynamic, list mode emission scan in the 3D mode, lasting 66 min, was undertaken [12, 13]. All patients had baseline diagnostic contrast-enhanced chest and abdomen CT performed within 1 month of study enrolment and restaging CT scanning after three cycles of chemotherapy. If available, baseline [^18^F]FDG-PET/CT were retrieved, and standardised uptake value (SUV)_mean_ and SUV_max_ of primary tumour reported.

### Image analysis

Raw PET data were corrected for scatter and attenuation and reconstructed with an iterative algorithm consisting of 8 iterations and 21 subsets. The data were binned into time frames as follows: 1* 30 (background), 6* 10, 4* 20, 4* 30, 5* 120, 4* 180 and 4* 600 s. The KSF was applied to the dynamic PET data. Decay-corrected images (unfiltered and filtered) were then viewed using the Analyze® software (Analyze Version 11; Biomedical Imaging Resource, USA) in order to assess the utility of the KSF in improving visualisation of tumours. The attenuation corrected PET images and CT data were fused and analysed on a dedicated workstation (Hermes diagnostics, Sweden) by a dual-trained radiologist/nuclear medicine physician as well. The physician was blinded to patient Ki-67, patient response and survival outcome at the time of analysis. All SUV analyses were conducted using PET uptake parameters generated on Hermes.

Gastro-oesophageal and metastatic lesions were defined as target lesions by RECIST 1.1 on CT [14]. The lesions on the [^18^F]FLT-PET/CT corresponding to those on the CT showing an increased uptake and visualised on both the unfiltered and the filtered images were considered as target lesions. The diameter of the target lesions was measured using electronic callipers on the PACS workstation. The same target lesions were used for analyses on both the PET/CT and CT, before and after treatment. In patients with multiple lesions, the sum of the parameters of all the lesions was calculated and change with treatment documented [15].

Consecutive regions of interest (ROIs) were manually defined around the tumours on the summed images, employing the patient’s diagnostic CT scan and the filtered images. The ROI encompassed the whole tumour for SUV analysis. The [^18^F]FLT radioactivity concentration within the ROIs was then normalised for injected radioactivity and body weight (grams) to obtain the mean and maximum SUV at 60 min (SUV60_mean_ and SUV60_max_) on baseline and post-treatment [^18^F]FLT-PET/CT (unfiltered) studies. The percentage change in SUV in both SUV_mean_ and SUV_max_ was then calculated for each target lesion visible on baseline imaging as (SUV_post_ – SUV_pre_)/SUV_pre_. The SUV_mean_ time activity curve was used to calculate the area under the curve (AUC) (kg min mL^−1^) from 0 to 1 min, reflecting tracer delivery to the tumour, as intimated in canine studies [16]. The SUVratio, 60:1 (SUV_mean_ 60 min/SUV_mean_ 1 min) was calculated as an indicator of tracer retention within the tumour. All target lesions were included in the final analysis.

### Immunohistochemistry

To evaluate the relationship between PET parameters and direct measurement of proliferation, paraffin-embedded tumour samples obtained from diagnostic specimens within 1 month preceding the PET scan were sectioned and immunostained with an anti-Ki-67 antibody (clone K-2) (Leica Biosystems, Wetzlar, Germany). The number of total and Ki-67-positive cells were manually counted in eight randomly selected fields of view using a BX51 Olympus microscope (Olympus Optical, Tokyo, Japan) at ×400 magnification and with the aid of Sigma Scan Pro 5 (Aspire Software International, Leesburg, VA). The Ki-67 proliferation index was calculated as the ratio of the number of Ki-67-positive cells to the total number of cells.

### Statistical analysis

Wilcoxon rank test was used to assess for differences between PET parameters and Ki-67 with clinical outcomes. The percentage change in SUV in both SUV_mean_, SUV_max_ SUVratio, 60:1 and AUC were plotted against clinical outcomes. A paired *t* test was used to assess the difference in these parameters over time. As the sample size was small for analysis purposes, complete and partial responses were grouped together as response, and stable disease and progressive disease was classed as non-response. Moreover, where patients had more than one target lesion, the median change in SUV_mean_ and SUV_max_ of all lesions was reported. Survival figures were calculated from date of diagnosis to date of death or date of last follow-up. A *p* ≤ 0.05 was considered significant. Statistical analysis was done using SPSS statistical package version 22 (SPSS Inc., Chicago, IL, USA).

## Results

### Patients

Ten patients, all of whom were male, were included (median age 65 years, range 54–76 years). Patient characteristics are summarised in Table 1. Four patients received chemotherapy prior to surgical resection, and six received palliative therapy (combination therapy with epirubicin and capecitabine, with either cisplatin or oxaliplatin). Four patients underwent oesophagectomy post three cycles of chemotherapy. No patient received radiotherapy during the study period. One patient was lost to follow-up and did not have formal RECIST assessment. The median time between baseline [^18^F]FLT-PET/CT and subsequent PET scan was 24.5 days (range 7–35).

**Table 1:** Characteristics of patients enrolled in study

Pt No	Age (years)	Site^a^	Stage^b^	Chemotherapy^c^	Overall RECIST response^d^	SUV_60, ave_ (preRx)	SUV_60, max_ (preRx)	Percentage Change SUV_60, ave_	Percentage Change SUV_60, max_	Baseline FDG SUV_60, ave_	Baseline FDG SUV_60,max_	Ki-67 (%)
1	63	GEJ	T4N1M1	EOX	PR	5.92	9.45	−35.16	−54.49	–	–	43
2	54	Distal	T3N0	ECX, surgery	–	4.6	4.52	−25.65	−5.75	5.93	4.38	–
3	68	Distal	T3N0M1	EOX	SD	5.32	7.47	−2.26	4.95	6.76	5.63	87
4	76	GEJ	T3N1M1	ECX	PR	4.97	7.25	6.44	5.66	21.45	19.96	29
5	71	GEJ	T3N0M0	EOX	SD	6.08	9.96	11.51	11.14	14.91	14.06	23
6	64	GEJ	T4N1M1	EOX	SD	5.5	8.85	−30.73	−47.57	10.44	9.03	–
7	65	Gastric	T3N1M0	EOX, Surgery	PR	4.14	7.62	−15.22	−16.93	–	–	–
7^e^						3.65	8.87	1.92	29.76			
8	60	Distal	T3N1M0	ECX, surgery	SD	7.7	13.36	−29.61	−33.08	–	–	–
9	54	Gastric	T3N1M0	EOX/STO3	SD	3.66	5.93	−24.86	−25.46	–	–	51
10	69	Distal	T3N0M0	ECX, surgery	SD	4.79	9.3	−18.99	−24.09	13.88	12.24	67.5

^a^ *GEJ* gastro-oesophageal junction, *distal* distal oesophagus

^b^Stage according to TNM criteria, CT and EUS used

^c^ *EOX* epirubicin, oxaliplatin and capecitabine, *ECX* epirubicin, cisplatin and capecitabine, ST03 study - ECX +/- bevacizumab

^d^RECIST criteria: *PR* partial response, *SD* stable disease, *PD* progressive disease

^e^Lymph node >20 mm, included as a target lesion in final analysis

### Imaging characteristics of [^18^F]FLT uptake at baseline

**Fig. 1:**
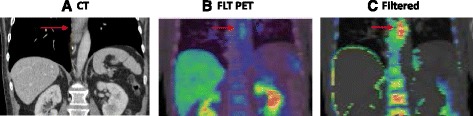
Coronal section of pre-treatment of a patient with oesophageal cancer (*arrows*) illustrating the diagnostic CT (**a**) and the unfiltered fused PET images (**b**). Following application of the KSF, visualisation of the oesophageal cancer (*arrows*) was markedly improved (**c**)

**Fig. 2:**
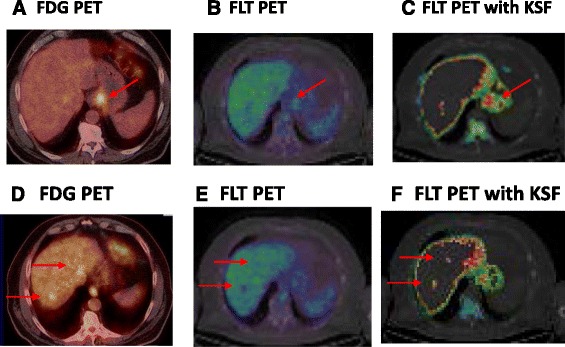
Transverse sections from a patient with gastroesophageal junction tumour with liver metastasis. On [^18^F]FDG-PET imaging, both the primary (**a**) and liver lesions (**d**) are visible. Whilst the primary oesophageal lesion is visible on the unfiltered [^18^F]FLT-PET imaging (**b**), visualisation is improved following application of the KSF (**c**). Liver metastases appear as an area of relative photopaenia (**e**) on [^18^F]FLT-PET imaging. Following application of the KSF, there is marked reduction of the background hepatic signal allowing for improved visualisation of hepatic metastases (**f**). Both lesions are also seen on baseline [^18^F]FDG-PET imaging (**d**)

On visual analysis, only six of the ten primary tumours were clearly visible on the unfiltered images. Following the application of KSF, image visualisation was improved in all the tumours as represented in Fig. 1a, b, and c such that all primary GOC were visualised on the filtered [^18^F]FLT-PET/CT images, enabling accurate assessment of the tumour. ROIs were drawn on the filtered images, with reference to baseline CT scans. Since KSF is associated with removal of delivery components within the data, we observed a mean (±SD) reduction in SUV_mean_ in untreated primary tumours of 12.4 % (±35.4) whilst the background signal in the liver was reduced to ~0. One patient had FLT avid lymph nodes, and one patient had evidence of liver metastases not seen on staging non-contrast CT imaging but demonstrated on baseline [^18^F]FDG-PET/CT and filtered [^18^F]FLT-PET/CT. The application of the KSF reduced background normal liver activity leading to improved visualisation of liver metastases in this case (Fig. 2b, c, e, and f).

All primary tumours and lymph nodes were included in analysis. The liver metastases were not included in analysis as these were not visible on the patient’s baseline non-contrast CT imaging for RECIST 1.1 measurement. The mean unfiltered SUV_60,max_ (±SD), SUV_60,mean_ (±SD) for the primary tumours on the baseline scan were 8.42 (±2.31) and 5.06 (±1.16).

As [^18^F]FLT is a biomarker of cellular proliferation, we investigated the relationship between Ki-67, a histologic marker of proliferation with the PET variables SUV_mean_ and SUV_max_. Histology was available for six patients, three samples were inadequate for Ki-67 examination and 1 tissue block was unavailable. A significant association was observed between Ki-67 and SUV_max_ (Z −2.01, *p* = 0.04) and SUV_mean_ (Z −2.02, *p* = 0.04) at baseline. No association was observed between Ki-67 and response to chemotherapy.

### Comparison between [^18^F]FDG and [^18^F]FLT

Pre-therapy [^18^F]FDG-PET/CT was performed for the six patients with oesophageal cancer, as part of clinical routine management. All primary tumours were visible both on baseline [^18^F]FDG-PET/CT and [^18^F]FLT-PET/CT.

The SUV_60,max_ of [^18^F]FLT of the primary tumours was lower than that of [^18^F]FDG (8.4 ± 2.3 vs. 12.2 ± 5.8; *p* = 0.18) as was SUV_60, mean_ (5.07 ± 1.2 vs. 10.8 ± 5.8; *p* = 0.07). One patient had three liver metastases seen on [^18^F]FDG-PET/CT. These were all detected as areas of relative photopaenia on [^18^F]FLT-PET images. Following application of the KSF, background liver uptake was reduced and metastases were better visualised (Fig. 2a–f). There was no association observed between baseline [^18^F]FDG tumour SUV_max_ and Ki-67 (*p* = 0.07).

### Effect of treatment on PET variables

**Fig. 3:**
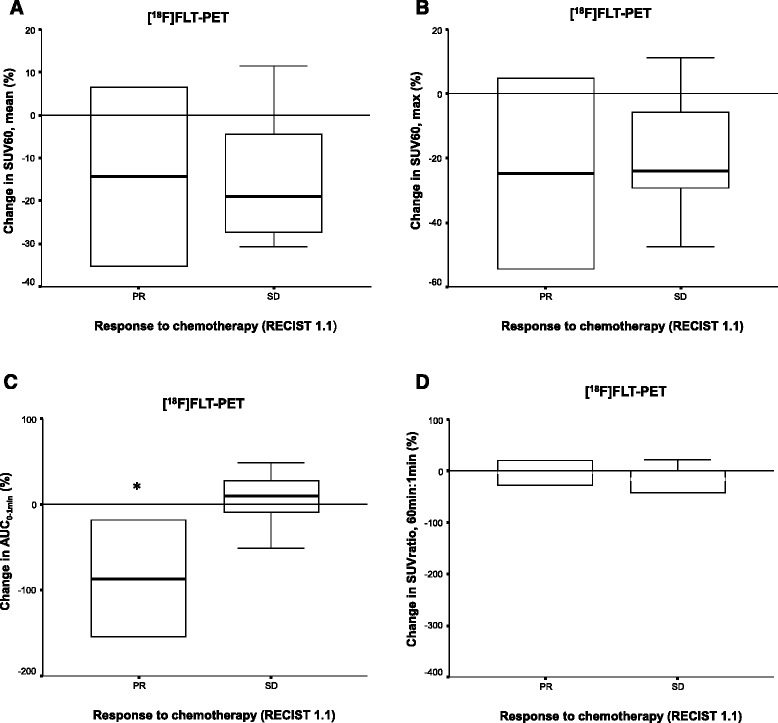
*Box plots* showing association between change in [^18^F]FLT-PET SUV_60, mean_ (**a**) and [^18^F]FLT-PET SUV_60, max_ (**b**) following one cycle of chemotherapy, and [^18^F]FLT-PET AUC_0–1 min_ (**c**), and [^18^F]FLT-PET SUV 60 min: 1 min (**d**) and response to chemotherapy after three cycles of chemotherapy (RECIST 1.1). *p < 0.05

According to RECIST 1.1, six patients had stable disease (SD), three patients had partial response (PR) following three cycles of chemotherapy, and one patient was lost to follow-up. There was a median overall reduction in [^18^F]FLT-PET/CT SUV_60, mean_ (−14.7 ± 16.4%) and SUV_60, max_ (−14.2 ± 25.9 %) following the first cycle of chemotherapy. No significant changes were observed in either [^18^F]FLT-PET/CT SUV_60, mean_ (Fig. 3a) or SUV_60, max_ (Fig. 3b) following one cycle of chemotherapy. Of the six patients with [^18^F]FDG-PET/CTs, there was no association noted between response and baseline SUV uptake.

In terms of [^18^F]FLT-PET/CT AUC_0–1 min_, there was a 8.3% (±47.4%) reduction following one cycle of treatment, and a significant association was observed between percentage change in AUC_0–1 min_ following one cycle of therapy and response (*p* = 0.047) (Fig. 3c), such that patients responding to chemotherapy had a greater reduction in AUC_0–1 min_ suggesting a significant reduction in tracer delivery. A reduction of 12.5% (±31.9%) in SUVratio, 60:1 was observed in the data set; however, no significant association was observed with response (Fig. 3d). The median progression-free survival was 24.6 months (95% CI 18.2–31.1), and overall survival had not been reached. Due to small numbers, no statistical analyses between PET parameters and survival were conducted.

## Discussion

The primary aim of this pilot study was to investigate the utility of the KSF when applied to [^18^F]FLT-PET/CT to improve visualisation of GOC. As a secondary endpoint, we explored the use of [^18^F]FLT-PET/CT as an early biomarker of clinical response compared to RECIST 1.1 following three cycles of chemotherapy. We have shown that application of the spatial filter does improve visualisation of both primary tumours and liver metastases, allowing more accurate localisation and therefore more accurate assessment of PET response.

The KSF has been investigated by our group in visualising pancreatic cancer, and liver metastases from different primary tumour sites, and we have consistently demonstrated the applicability of this technique [17, 18]. As illustrated in Figs. 1 and 2, implementation of the KSF removed physiological background activity allowing improved lesion detection. Only six of the ten primary tumours were visible above background on the unfiltered data. The application of the filter delineated all primary tumours and allowed improved detection of the liver metastases in one patient. This is of particular importance in the neoadjuvant setting where accurate staging determines which patients will undergo surgery. [^18^F]FLT imaging is useful particularly in the differentiation between tumour and inflammation, a major concern given the majority of oesophageal cancers will develop on a background of Barrett’s oesophagus [19]. Moreover, in gastric cancer, there is limited utility of [^18^F]FDG-PET. However, despite the interest in [^18^F]FLT-PET imaging, its utility has been limited by high physiologic uptake within the liver. Application of the KSF opens the clinical arena of [^18^F]FLT-PET imaging in upper gastrointestinal tumours by improving tumour visualisation with the hope of improving patient outcome by improved detection and differentiation of both primary lesions and metastases.

As a translational endpoint, we described the association between Ki-67 and [^18^F]FLT imaging parameters, a finding well described in other tumour types [20]. However, there have only been three studies in GOC tumours none of which demonstrate a relationship between [^18^F]FLT uptake and Ki-67 [7, 8, 21]. Differing results may be attributed to the small sample size of the published studies, and tumour heterogeneity, particularly as both in this study and others, correlation between SUV uptake at the actual site of the biopsy was not conducted. We anticipated a significant association between Ki-67 and [^18^F]FLT uptake given that [^18^F]FLT is a marker of proliferation, being a substrate for the cell cycle regulated enzyme TK-1, an enzyme which is up-regulated in proliferating cells. In a reaction mediated by TK-1, [^18^F]FLT is phosphorylated to [^18^F]FLT-monophosphate which is not incorporated into the DNA but remains trapped within the cytosol where it accumulates, thereby acting as a marker of proliferation [22].

Early evaluation of treatment response to chemotherapy with [^18^F]FLT-PET has been evaluated in a number of tumour types, where [^18^F]FLT-PET response as early as 1 week following chemotherapy has been shown to predict treatment outcome [23, 24]. Even though there have been a number of studies comparing the sensitivity and specificity of both [^18^F]FLT-PET and [^18^F]FDG-PET in detecting oesophageal cancer, there has only been one study in this disease type investigating [^18^F]FLT-PET as an early response biomarker following induction chemotherapy with oxaliplatin and S-1 prior to radiotherapy and resection [10]. The authors report a significant reduction in [^18^F]FLT uptake in patients responding to chemotherapy compared to non-responders following two cycles of chemotherapy [10]. However, only nine patients were evaluable, only one of which did not respond to therapy and these results need further validation. Two further studies illustrate dramatic reduction in [^18^F]FLT uptake in tumours 4 weeks following radiotherapy, consistent with clinical response [25, 26]. In the study by Yue and colleagues, the authors also conclude that [^18^F]FLT-PET may be useful in differentiating between residual tumour and inflammation, post-radiotherapy, a key consideration in terms of determining the efficacy of chemoradiotherapy. In our study, all oesophageal lesions were visible by [^18^F]-FDG-PET. This is not an unexpected finding since all the primary tumours were adenocarcinoma and it is known that this histological subtype has high [^18^F]-FDG uptake [27].

Given the variable sensitivity of FDG in imaging gastric cancer, [^18^F]FLT-PET has been investigated in a number of studies, again predominantly comparing the efficacy of [^18^F]FLT-PET and [^18^F]FDG-PET [8, 9, 28, 29]. The largest study is that by Wang and colleagues who enrolled 64 patients with advanced gastric cancer to compare the sensitivity and specificity of [^18^F]FLT-PET and [^18^F]FDG-PET in predicting both response to chemotherapy and survival. The authors conclude that whilst [^18^F]FDG-PET uptake predicted both response and survival, [^18^F]FLT-PET showed little utility, particularly with regard to assessment of liver metastases, where background hepatic uptake precluded any assessment of treatment response. Ott and colleagues investigated [^18^F]FLT-PET as a response marker to neoadjuvant chemotherapy in patients with gastric cancer (*n* = 45) and reported a significant association between SUV_mean_ following one cycle of chemotherapy (2 weeks after the initiation of therapy) and prognosis, but not treatment response [7].

Whilst the SUVs obtained in our study in both oesophageal and gastric cancers were consistent with those published, we did not observe any significant association between [^18^F]FLT SUV and response to treatment, despite the improvement in tumour visualisation with the application of the KSF. Because early changes in perfusion have been shown to associate with therapy response in oesophageal and gastric cancer, we assessed potential [^18^F]FLT delivery variables embodied within AUC_0–1 min_ [30]. The utility of AUC_0–1 min_ has not been validated in humans; however, its application has been validated in a canine tumour model [14]. We therefore, chose to apply this PET parameter to our dataset. [^18^F]FLT SUV has been shown to be highly correlated to vascular fraction and perfusion/permeability soon after tracer injection [16]. Differences in [^18^F]FLT delivery, as indicated by AUC_0–1 min_, was coincident with treatment response such that patients responding to therapy had a reduction in AUC_0–1 min_. This can be interpreted as a reduction in the delivery of [^18^F]FLT to the tumour following chemotherapy in responding patients analogous to CT perfusion studies in the same group of patients [30]. A perfusion deficit in responding tumours [30] will likely confound measurements of [^18^F]FLT retention. Taken together, therefore, the results of this pilot study would suggest that future studies of [^18^F]FLT-PET in this patient population may be of limited value.

The main limitation of this pilot study is the small sample, and mixed tumour type, both in terms of tumour staging and primary sites. Furthermore, patients received continuous capecitabine which has been shown in breast cancer to cause [^18^F]FLT “flare” resulting from translocation of equilibrative nucleoside transporter 1 (ENT1) to the cellular membrane and consequently an increase in [^18^F]FLT uptake [31, 32]. This “flare response” was observed 24–48 h post-capecitabine therapy. However, it is clear from the dynamic analyses that there is no flare in the post-treatment scans. Another issue is in the analysis where in the one patient with more than one target lesion, the average of SUV was taken across both target lesions. This methodology does not take into account therefore, heterogeneity between the primary and metastatic deposit. Despite these limitations, the main strength of this study is that by conducting dynamic [^18^F]FLT scanning in this patient population, we have been able to evaluate and demonstrate treatment-related delivery changes.

## Conclusions

We have shown that application of a KSF allows accurate visualisation of both primary and metastatic gastro-oesophageal cancer following imaging with the proliferation marker, [^18^F]FLT-PET/CT. However, [^18^F]FLT-PET uptake parameters did not correlate with response. Instead, we observe significant changes in tracer delivery following chemotherapy suggesting that further [^18^F]FLT-PET/CT studies in this tumour type should be undertaken with caution.
